# Association of white blood cell count to hemoglobin ratio with the life quality after laparoscopic surgery in patients with endometriosis

**DOI:** 10.3389/fendo.2025.1655476

**Published:** 2025-10-13

**Authors:** Weiwei Shen, Huan Chen, Xiaoming Zhou, Yichen Chen, Jue Zhu, Jing Zhang

**Affiliations:** ^1^ Department of Gynecology, The First Affiliated Hospital of Ningbo University, Ningbo, Zhejiang, China; ^2^ Department of Gynecology, Women and Children’s Hospital of Ningbo University, Ningbo, Zhejiang, China; ^3^ Basic Research Laboratory, Women and Children’s Hospital of Ningbo University, Ningbo, Zhejiang, China; ^4^ Ningbo University, Ningbo, Zhejiang, China

**Keywords:** WHR, endometriosis, life quality, laparoscopic surgery, SF-36

## Abstract

**Background:**

Endometriosis (EM) is a common hormone-dependent and chronic inflammatory disease affecting women of reproductive age, characterized by pelvic pain, infertility, and reduced quality of life. Laparoscopic surgery is a primary treatment, yet the influence of preoperative factors on postoperative outcomes remains unclear. The white blood cell count to hemoglobin ratio (WHR), a novel marker of systemic inflammation and tissue hypoxia, has shown prognostic value in surgical oncology but its role in predicting postoperative quality of life in EM patients remains to be elucidated.

**Objective:**

To explore association of white blood cell count to hemoglobin ratio (WHR) with the life quality after laparoscopic surgery in patients with endometriosis (EM).

**Methods:**

Data on 271 EM patients were extracted from The First Affiliated Hospital of Ningbo University in December 2016 to October 2022. Multivariate linear regression analyses were utilized to investigate the associations of WHR levels with eight health concepts in the Short-Form 36 (SF-36) scale (3-month postoperative evaluation) and evaluated through β with 95% confidence intervals (CIs). Subgroup analyses of age, body mass index (BMI), clinical stage, pathological classification, pelvic pain and lysis of adhesion were also performed. Given <1% covariate missingness, analyses used complete cases; multiple imputation would be unlikely to change the conclusions.

**Results:**

Patients were divided into WHR tertiles (<0.04, 0.04-0.05, ≥0.05) for description; primary models treated WHR as continuous. Higher WHR was associated with lower SF-36 scores: PCS (β = -1.42, 95% CI -2.20 to -0.65), PF (-2.82, -4.06 to -1.58), RP (-2.21, -3.94 to -0.49), VT (-2.00, -3.59 to -0.41) and RE (-2.41, -4.23 to -0.59). Tertile contrasts showed similar patterns (raw P<0.05). After BH-FDR, the WHR-PCS association remained in age<40, BMI<24 kg/m², stage IV, ovary/peritoneal phenotype, no pelvic pain, and left/right adhesion + rectum-vaginal adhesion (q<0.05); other subgroups are exploratory.

**Conclusion:**

Higher pre-operative WHR was associated with lower PCS at 3 months post-surgery. FDR-adjusted analyses supported the association in selected subgroups, while other contrasts were exploratory. Pending external validation and clinically meaningful cut-offs, WHR may complement existing factors for postoperative risk stratification.

## Background

Endometriosis (EM) is a chronic condition in women of childbearing age that affects about one tenth women and other individuals with a uterus worldwide, causing a huge burden of diseases (1). The most common symptoms of EM are pelvic pain and infertility, and it have significant effects on quality of life, fertility, and risk of malignancy (2). Laparoscopic surgery is the preferred first-line modality of diagnosis and treatment of EM that significantly influences multiple dimensions of quality of life in EM patients (3). However, evidence on the influence of preoperative related factors on the postoperative quality of life in EM patients is still limited (4).

Inflammation plays an important role in the development and pain of EM, and it is also a crucial factor affecting the prognosis of surgical patients (5, 6). In addition to inflammation, anemia is also an important factor affecting the prognosis of surgical patients and is closely related to inflammation (7, 8). Recently, white blood cell count to hemoglobin ratio (WHR) has been developed to characterize immune inflammatory states and anoxic microenvironments and has been found to be a prognostic factor for cancer patients underwent surgery (9–11). Nevertheless, no study has discussed the role of WHR in the quality of life among EM patients after laparoscopic surgery.

Herein, this study aims to investigate the association between WHR and the quality of life in different dimensions after laparoscopic surgery among EM patients, and to provide some information for clinical management and decision-making in this population.

## Methods

### Study design and participants

This single-center retrospective cohort study was conducted at The First Affiliated Hospital of Ningbo University in December 2016 to October 2022. Women aged ≥18 years with endometriosis (EM) diagnosed by laparoscopic surgery met the inclusion criteria. The exclusion criteria were (1) received open surgery or open surgery combined with laparoscopic surgery, (2) underwent hysterectomy or uterus with bilateral adnexectomy, (3) missing information on white blood cell count (WBC), hemoglobin (HB) or quality of life evaluation, (4)Active infection at the pre-operative assessment (any of: temperature ≥38.0 °C within 48 h, C-reactive protein >10 mg/L or procalcitonin ≥0.5 ng/mL, a new clinical focus consistent with infection, or systemic antibiotics initiated for an acute infection, including suspected pelvic inflammatory disease), (5) Pregnancy or ≤6 weeks postpartum/lactation. A total of 271 patients were included in the final analysis. The study complied with the Declaration of Helsinki; written informed consent was obtained and the protocol was approved by the institutional ethics committee.

To ensure a clear temporal sequence, the exposure (WHR) was measured within 24 hours before surgery, and the outcome (SF-36) was administered post-operatively (details below). Because WHR preceded the outcome assessment, the study is labelled a retrospective cohort; in a sensitivity analysis we treated any subset with non-pre-operative sampling as cross-sectional.

### Definitions and outcomes

The white-blood-cell-to-hemoglobin ratio (WHR) was calculated as WBC (×10^9^/L) divided by Hb (g/L). For descriptive balance and visualization—without imposing a disease-agnostic clinical threshold—WHR was secondarily grouped into tertiles: <0.04, 0.04-0.05, and ≥0.05. Note that WHR thresholds published in oncology often use a different computational convention—WBC (per mm³)/[10 × Hb (g/L)]—which is numerically ~100× our definition (WBC (×10^9^/L)/Hb (g/L)). Consequently, published oncology cut-offs such as 2.855 and 4.604 correspond to approximately 0.0286 and 0.0460 in our units and are not directly transportable to endometriosis; therefore, tertiles in this study are used for description only (10) (12).

The primary outcome was the Short-Form 36 (SF-36) Health Survey for 3 months (follow-up duration was 3 months) after the laparoscopic surgery. The SF-36 questionnaire includes 36 items measuring eight health concepts: physical functioning, role-physical, bodily pain, general health, vitality, social functioning, role-emotional, and mental health. The score range of each of the eight dimensions is from 0 to 100, and the higher the score, the better the health condition. Additionally, the total Physical Health Score (PCS) and the total Mental Health Score (MCS) summarize the above eight dimensions into two parts:


SF‐36 PCS=Σ(z‐score of each scale×respective physical factor coefficient)×10+50

SF‐36 MCS=Σ(z‐score of each scale×respective mental factor coefficient)×10+50

### Covariates

Baseline characteristics included age, body mass index (BMI), revised ASRM (rASRM) stage, pathological classification/phenotype, recurrence status, pelvic pain (yes/no), lysis of adhesion (yes/no), gynecologic disease history, parity/childbearing history, AST/ALT ratio, albumin (ALB), creatinine (Cr) and fibrinogen (FIB).

### Laboratory assays

Venous blood was drawn within 24 hours before surgery. WBC and Hb were measured on [XN9000, Sysmex, Japan] under routine internal quality control; analytical precision was verified by daily internal QC and remained within manufacturer specifications; published evaluations report low CV% (generally ≤1% for routine blood parameters) on this platform.

### Missing data

Overall missingness was very low (≤0.74%) and confined to secondary laboratory covariates (creatinine, albumin, AST/ALT; **Supplementary Table S1**); the exposure (WHR) and the outcomes (SF-36) were complete. We therefore prespecified complete-case analysis (CCA) as the primary approach to avoid unnecessary model-based assumptions (13). As a sensitivity analysis, we applied multiple imputation by chained equations (MICE; m=20) under a missing-at-random (MAR) assumption, including all covariates and outcomes in the imputation model; convergence diagnostics were satisfactory. Estimates pooled with Rubin’s rules were highly consistent with the CCA results (**Supplementary Table S2**), supporting the robustness of our inferences.

### Statistical analysis

Continuous variables were summarized as mean ± SD if approximately normal or median (Q1, Q3) otherwise; categorical variables as n (%). Group differences across WHR tertiles were assessed using one-way ANOVA (or Kruskal–Wallis for non-normal distributions) and χ² tests (or Fisher’s exact test when expected counts were <5).

Associations between WHR and SF-36 outcomes (PCS, MCS and the eight domains) were evaluated using linear regression, reporting β coefficients with 95% confidence intervals (CIs) and two-sided P-values. Model 1 was unadjusted. Model 2 was adjusted *a priori* for age, BMI, rASRM stage, pathological classification/phenotype, recurrence, pelvic pain, lysis of adhesion, gynecologic history, parity, AST/ALT ratio, albumin, creatinine and fibrinogen. Primary inference was based on the cohort-level models with a significance threshold of P<0.05.

Subgroup analyses were pre-specified for age, BMI, rASRM stage, phenotype, pelvic pain and adhesiolysis. To mitigate false positives from multiple comparisons, we controlled the false discovery rate (FDR) using the Benjamini–Hochberg procedure within each subgroup family and report q-values alongside raw P-values in the subgroup tables; q<0.05 was considered statistically significant for subgroups (q <0.10 considered suggestive).

WHR tertiles were used for descriptive balance and presentation only; primary modelling treated WHR as a continuous exposure. All analyses were conducted in R, version 4.3.1 (R Foundation for Statistical Computing, Vienna, Austria).

Causal assumptions are detailed in **Supplementary Methods** and depicted in **Supplementary Figure**.

## Results

### Characteristics of patients with EM

**Figure 1:**
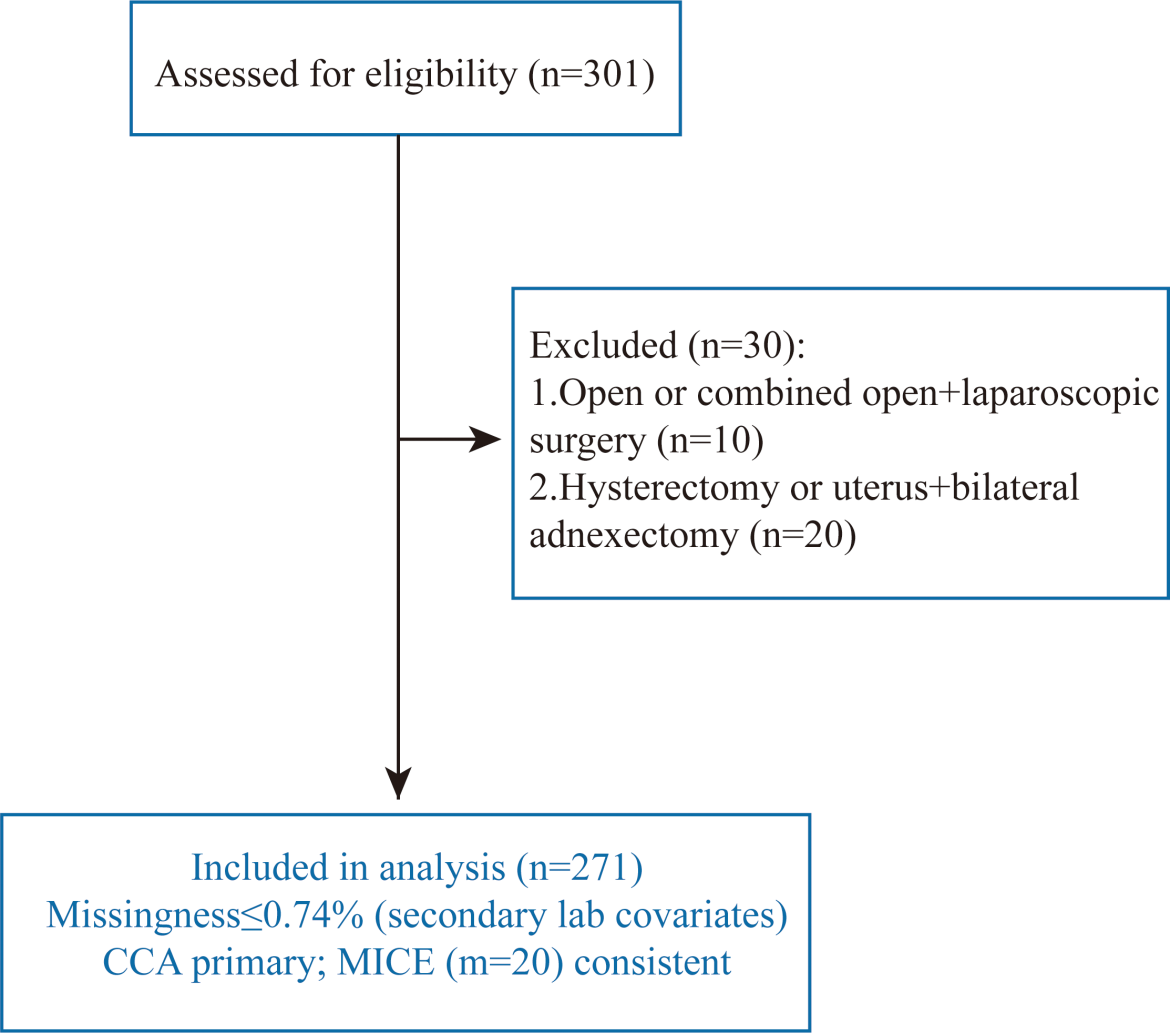
Study flow.

Of 301 women assessed for eligibility, 30 were excluded (open or combined open + laparoscopic surgery, n=10; hysterectomy or uterus + bilateral adnexectomy, n=20), leaving 271 for analysis. Missingness was ≤0.74% and confined to secondary laboratory covariates. The primary analysis used complete cases; multiple imputation (MICE, m=20) yielded consistent estimates (**Figure 1**). Missingness was ≤0.74% for laboratory covariates; imputed and complete-case results were consistent (**Supplementary Tables S1**-**S2**).

Comparation on characteristics of eligible patients with different WBC/HGB levels was shown in **Table 1**. The average age of total patients was 36.49 years old. Among the three groups, patients with WHR level of ≥0.05 had the highest average BMI values (22.46 kg/m^2^), followed by those with WHR level of 0.04-0.05 (average BMI = 21.68 kg/m^2^). More than half of participants were at EM clinical stages of I/II/III (n=155, 57.20%), had pathological classification of ovary/peritoneal (n=182, 67.16%). EM patients with WHR level of ≥0.05 had the highest average levels of ALB (43.29 g/L), and the lowest levels of physical functioning (88.01), role-physical (89.89), PCS (53.85), vitality (88.15), social functioning (86.07) and role-emotional (91.75) scores. 

**Table 1:** Characteristics of EM patients in different WHR level groups.

Variables	Total (n=271)	WHR			Statistics	*P*
		<0.04 (n=90)	0.04-0.05 (n=92)	≥0.05 (n=89)		
Age, years, Mean ± SD	36.49 ± 7.47	36.30 ± 8.42	35.61 ± 6.91	37.61 ± 6.94	F=1.671	0.190
BMI, kg/m^2^, Mean ± SD	21.77 ± 3.26	21.17 ± 3.23	21.68 ± 3.07	22.46 ± 3.39	F=3.621	0.028
BMI, kg/m^2^, n (%)					-	0.050
<18.5	39 (14.39)	20 (22.22)	10 (10.87)	9 (10.11)		
[18.5, 24)	177 (65.31)	55 (61.11)	68 (73.91)	54 (60.67)		
[24, 28)	44 (16.24)	11 (12.22)	12 (13.04)	21 (23.60)		
≥28	11 (4.06)	4 (4.44)	2 (2.17)	5 (5.62)		
Clinical stages, n (%)					χ^2^=0.388	0.824
I/II/III	155 (57.20)	50 (55.56)	55 (59.78)	50 (56.18)		
IV	116 (42.80)	40 (44.44)	37 (40.22)	39 (43.82)		
Pathological classification, n (%)					χ^2^=1.782	0.410
Ovary/peritoneal	182 (67.16)	62 (68.89)	57 (61.96)	63 (70.79)		
Ovary/peritoneal combined with deep infiltration	89 (32.84)	28 (31.11)	35 (38.04)	26 (29.21)		
Recurrence EM, n (%)					χ^2^=0.242	0.886
No	250 (92.25)	83 (92.22)	84 (91.30)	83 (93.26)		
Yes	21 (7.75)	7 (7.78)	8 (8.70)	6 (6.74)		
Pelvic pain, n (%)					χ^2^=0.773	0.679
No	180 (66.42)	62 (68.89)	62 (67.39)	56 (62.92)		
Yes	91 (33.58)	28 (31.11)	30 (32.61)	33 (37.08)		
Lysis of adhesion, n (%)					-	0.509
No	3 (1.11)	1 (1.11)	1 (1.09)	1 (1.12)		
Only left/right adhesion	106 (39.11)	37 (41.11)	30 (32.61)	39 (43.82)		
Only rectum-vagina	1 (0.37)	1 (1.11)	0 (0.00)	0 (0.00)		
Left/right adhesion combined with rectum-vagina	161 (59.41)	51 (56.67)	61 (66.30)	49 (55.06)		
History of gynecological diseases, n (%)					χ^2^=11.846	0.296
No	102 (37.64)	39 (43.33)	33 (35.87)	30 (33.71)		
Gynecological surgery	64 (23.62)	16 (17.78)	25 (27.17)	23 (25.84)		
Myoma of uterus	26 (9.59)	8 (8.89)	10 (10.87)	8 (8.99)		
Adenomyosis	6 (2.21)	0 (0.00)	4 (4.35)	2 (2.25)		
Infertility	21 (7.75)	10 (11.11)	5 (5.43)	6 (6.74)		
Multiple diseases	52 (19.19)	17 (18.89)	15 (16.30)	20 (22.47)		
Childbearing history, n (%)					χ^2^=6.549	0.365
No	75 (27.68)	30 (33.33)	22 (23.91)	23 (25.84)		
Full-term birth	71 (26.20)	23 (25.56)	28 (30.43)	20 (22.47)		
Abortion	16 (5.90)	6 (6.67)	7 (7.61)	3 (3.37)		
Multiple types	109 (40.22)	31 (34.44)	35 (38.04)	43 (48.31)		
AST/ALT, M (Q_1_, Q_3_)	1.60 (1.25, 2.00)	1.53 (1.23, 1.88)	1.59 (1.26, 2.05)	1.64 (1.29, 1.92)	χ^2^=1.941#	0.379
ALB, g/L, Mean ± SD	44.01 ± 3.45	44.65 ± 3.11	44.07 ± 3.78	43.29 ± 3.30	F=3.552	0.030
Cr, mg/L, Mean ± SD	55.49 ± 7.91	55.00 ± 7.65	54.48 ± 7.24	57.04 ± 8.67	F=2.647	0.073
FIB, Mean ± SD	2.31 ± 0.73	2.26 ± 0.71	2.23 ± 0.67	2.44 ± 0.79	F=2.228	0.110
WBC, K/uL, Mean ± SD	5.70 ± 1.75	4.26 ± 0.59	5.47 ± 0.66	7.39 ± 1.89	F=155.399	<0.001
HB, g/L, Mean ± SD	124.24 ± 14.85	128.81 ± 11.77	124.89 ± 14.08	118.96 ± 16.78	F=10.705	<0.001
Physical functioning, Mean ± SD	91.14 ± 11.04	94.06 ± 6.70	91.30 ± 13.88	88.01 ± 10.53	F=7.017	0.001
Role-physical, Mean ± SD	92.44 ± 14.69	95.83 ± 10.76	91.58 ± 16.69	89.89 ± 15.41	F=3.992	0.020
Bodily pain, Mean ± SD	89.98 ± 14.43	92.98 ± 12.25	87.63 ± 17.11	89.37 ± 12.99	F=3.298	0.038
General health, Mean ± SD	89.67 ± 13.43	92.06 ± 8.67	88.75 ± 17.33	88.20 ± 12.57	F=2.185	0.114
PCS, M (Q_1_, Q_3_)	55.27 ± 6.96	56.97 ± 4.20	54.98 ± 8.98	53.85 ± 6.51	F=4.743	0.009
Vitality, Mean ± SD	90.06 ± 13.63	93.11 ± 9.93	88.91 ± 15.93	88.15 ± 13.89	F=3.525	0.031
Social functioning, Mean ± SD	88.08 ± 15.20	91.67 ± 10.41	86.52 ± 18.18	86.07 ± 15.42	F=3.847	0.023
Role-emotional, Mean ± SD	93.84 ± 15.30	97.40 ± 11.44	92.38 ± 17.21	91.75 ± 16.14	F=3.768	0.024
Mental health, Mean ± SD	90.58 ± 12.08	93.53 ± 6.06	88.87 ± 15.97	89.35 ± 11.56	F=4.169	0.016
MCS, M (Q_1_, Q_3_)	59.45 ± 6.70	61.04 ± 4.65	58.49 ± 8.10	58.85 ± 6.63	F=3.935	0.021

F: One-Way ANOVA, χ^2^: chi-square test.

EM, endometriosis; WHR, white blood cell to hemoglobin ratio; SD, standard deviation; BMI, body mass index, AST/ALT, glutamic oxaloacetic transaminase to serum glutamic pyruvic transaminase ratio; M; median; Q1, 1st quartile; Q3, 3rd quartile; ALB; albumin; Cr; creatinine; FIB; fibrinogen; WBC, white blood cell; PCS, Physical Health Score; MCS, Mental Health Score.

### Associations of WHR with the quality of life after laparoscopic surgery

**Figure 2:**
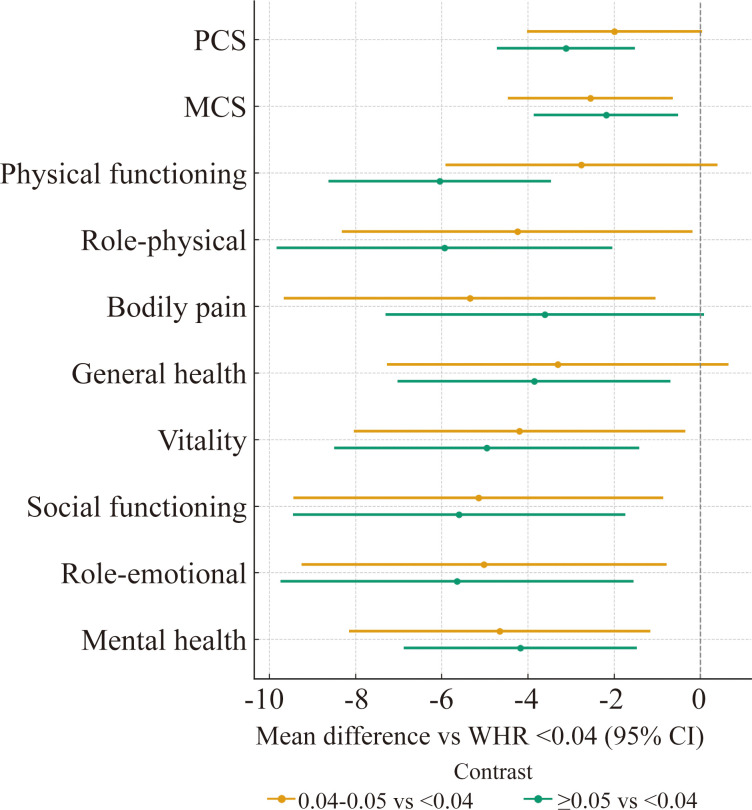
SF-36 domain scores by WHR tertile—forest plot of mean differences versus the reference group (WHR <0.04).

**Figure 2** summarizes domain-specific differences by WHR tertile relative to WHR <0.04. On average, the WHR <0.04 group had the highest scores across domains. Both the 0.04-0.05 and ≥0.05 groups showed lower mean SF-36 scores across most domains, with the largest decrements observed in physical functioning, role-physical, vitality and role-emotional. These descriptive patterns were directionally consistent with the multivariable models (**Table 2**), in which higher (continuous) WHR was associated with lower PCS, PF, RP, VT and RE. Multiplicity-adjusted q-values are provided in **Table 2** (and in **Tables 3**–**8** for prespecified subgroups).

**Table 2:** Association between WHR and life quality after laparoscopic surgery in EM patients.

Variables	Model 1			Model 2		
	β (95% CI)	*P*	*q*	β (95% CI)	*P*	*q*
PCS						
WHR	-1.65 (-2.46, -0.84)	<0.001	0.006	-1.42 (-2.20, -0.65)	<0.001	0.01
WHR levels	Ref			Ref		
<0.04	-2.13 (-4.15, -0.12)	0.039	0.045	-2.34 (-4.20, -0.48)	0.014	0.03
0.04-0.05	-2.95 (-4.95, -0.95)	0.004	0.02	-2.68 (-4.52, -0.84)	0.005	0.0214
≥0.05						
Physical functioning						
WHR	-3.14 (-4.40, -1.87)	<0.001	0.006	-2.82 (-4.06, -1.58)	<0.001	0.01
WHR levels						
<0.04	Ref			Ref		
0.04-0.05	-2.93 (-6.10, 0.24)	0.071	0.0761	-3.38 (-6.38, -0.38)	0.028	0.0494
≥0.05	-5.76 (-8.91, -2.62)	<0.001	0.006	-5.36 (-8.34, -2.39)	<0.001	0.01
Role-physical						
WHR	-2.90 (-4.62, -1.18)	0.001	0.006	-2.21 (-3.94, -0.49)	0.012	0.03
WHR levels						
<0.04	Ref			Ref		
0.04-0.05	-4.54 (-8.80, -0.28)	0.038	0.045	-5.01 (-9.11, -0.92)	0.017	0.034
≥0.05	-5.62 (-9.84, -1.39)	0.010	0.0273	-4.62 (-8.68, -0.56)	0.027	0.0494
Bodily pain						
WHR	-2.14 (-3.84, -0.43)	0.015	0.03	-1.59 (-3.23, 0.05)	0.059	0.0737
WHR levels						
<0.04	Ref			Ref		
0.04-0.05	-5.46 (-9.65, -1.27)	0.011	0.0275	-5.26 (-9.14, -1.38)	0.008	0.03
≥0.05	-3.55 (-7.71, 0.60)	0.095	0.095	-2.83 (-6.68, 1.02)	0.151	0.151
General health						
WHR	-1.82 (-3.41, -0.23)	0.026	0.0355	-1.35 (-2.92, 0.22)	0.094	0.1044
WHR levels						
<0.04	Ref			Ref		
0.04-0.05	-3.68 (-7.60, 0.23)	0.066	0.0733	-4.00 (-7.73, -0.28)	0.036	0.054
≥0.05	-3.47 (-7.36, 0.42)	0.081	0.0838	-3.13 (-6.82, 0.57)	0.099	0.1061
MCS						
WHR	-0.93 (-1.72, -0.13)	0.023	0.0343	-0.61 (-1.41, 0.19)	0.135	0.1397
WHR levels						
<0.04	Ref			Ref		
0.04-0.05	-2.64 (-4.58, -0.70)	0.008	0.0267	-2.78 (-4.66, -0.90)	0.004	0.0214
≥0.05	-2.12 (-4.05, -0.20)	0.032	0.04	-1.81 (-3.67, 0.05)	0.057	0.0737
Vitality						
WHR	-2.17 (-3.78, -0.56)	0.009	0.027	-2.00 (-3.59, -0.41)	0.014	0.03
WHR levels						
<0.04	Ref			Ref		
0.04-0.05	-4.40 (-8.36, -0.45)	0.030	0.0391	-5.01 (-8.78, -1.25)	0.010	0.03
≥0.05	-4.74 (-8.67, -0.82)	0.019	0.0316	-4.85 (-8.58, -1.11)	0.012	0.03
Social functioning						
WHR	-2.52 (-4.31, -0.72)	0.006	0.0257	-1.67 (-3.43, 0.09)	0.064	0.0768
WHR levels						
<0.04	Ref			Ref		
0.04-0.05	-5.26 (-9.67, -0.85)	0.020	0.0316	-5.96 (-10.10, -1.82)	0.005	0.0214
≥0.05	-5.47 (-9.84, -1.10)	0.015	0.03	-4.52 (-8.63, -0.42)	0.032	0.0505
Role-emotional						
WHR	-3.05 (-4.84, -1.26)	<0.001	0.006	-2.41 (-4.23, -0.59)	0.010	0.03
WHR levels						
<0.04	Ref			Ref		
0.04-0.05	-5.28 (-9.72, -0.84)	0.020	0.0316	-4.80 (-9.14, -0.46)	0.031	0.0505
≥0.05	-5.39 (-9.79, -0.99)	0.017	0.0316	-4.34 (-8.64, -0.03)	0.049	0.0668
Mental health						
WHR	-1.84 (-3.26, -0.41)	0.012	0.0277	-1.28 (-2.71, 0.15)	0.080	0.0923
WHR levels						
<0.04	Ref			Ref		
0.04-0.05	-4.86 (-8.36, -1.36)	0.007	0.0262	-5.06 (-8.42, -1.71)	0.003	0.0214
≥0.05	-4.01 (-7.48, -0.54)	0.024	0.0343	-3.54 (-6.87, -0.21)	0.038	0.0543

WHR, white blood cell to hemoglobin ratio; EM, endometriosis; CI, confidence interval; PCS, Physical Health Score; Ref, reference; MCS, Mental Health Score.

Model 1: unadjusted model,

Model 2: adjusted for age, BMI, clinical stage, pathological classification, recurrence, pelvic pain, lysis of adhesion, gynecological diseases history, childbearing history, AST/ALT, ALB, Cr and FIB.

**Table 3:** Association between WHR and life quality in age subgroups.

Variables	Age: <40			Age: ≥40		
	β (95% CI)	*P*	*q*	β (95% CI)	*P*	*q*
PCS						
WHR	-2.05 (-3.26, -0.84)	0.001	0.015	-1.21 (-2.19, -0.24)	0.017	0.114
WHR levels						
<0.04	Ref			Ref		
0.04-0.05	-3.49 (-5.96, -1.03)	0.006	0.0343	0.14 (-2.68, 2.96)	0.925	0.925
≥0.05	-3.69 (-6.37, -1.00)	0.008	0.0343	-2.28 (-4.82, 0.26)	0.083	0.195
Physical functioning						
WHR	-3.83 (-5.73, -1.93)	<0.001	0.015	-2.19 (-3.96, -0.42)	0.017	0.114
WHR levels						
<0.04	Ref			Ref		
0.04-0.05	-5.36 (-9.27, -1.45)	0.008	0.0343	0.99 (-4.07, 6.05)	0.703	0.7272
≥0.05	-6.73 (-10.99, -2.48)	0.002	0.02	-4.44 (-9.00, 0.11)	0.060	0.18
Role-physical						
WHR	-3.64 (-6.23, -1.06)	0.007	0.0343	-1.76 (-4.22, 0.69)	0.163	0.2965
WHR levels						
<0.04	Ref			Ref		
0.04-0.05	-5.53 (-10.79, -0.26)	0.042	0.09	-4.64 (-11.67, 2.39)	0.200	0.3333
≥0.05	-7.17 (-12.91, -1.44)	0.015	0.0562	-3.60 (-9.92, 2.72)	0.268	0.3655
Bodily pain						
WHR	-1.48 (-4.05, 1.09)	0.261	0.27	-2.29 (-4.35, -0.24)	0.032	0.1414
WHR levels						
<0.04	Ref			Ref		
0.04-0.05	-5.84 (-11.00, -0.68)	0.028	0.084	-3.66 (-9.63, 2.32)	0.235	0.3443
≥0.05	-3.12 (-8.74, 2.50)	0.279	0.279	-4.08 (-9.45, 1.30)	0.141	0.282
General health						
WHR	-1.99 (-4.44, 0.46)	0.114	0.1425	-0.84 (-2.68, 0.99)	0.371	0.4839
WHR levels						
<0.04	Ref			Ref		
0.04-0.05	-5.60 (-10.69, -0.51)	0.033	0.09	-1.21 (-6.48, 4.06)	0.653	0.6996
≥0.05	-5.51 (-11.05, 0.04)	0.053	0.0988	-1.91 (-6.65, 2.83)	0.433	0.5019
MCS						
WHR	-0.80 (-2.05, 0.45)	0.211	0.2344	-0.45 (-1.54, 0.65)	0.429	0.5019
WHR levels						
<0.04	Ref			Ref		
0.04-0.05	-2.17 (-4.69, 0.35)	0.093	0.1268	-4.29 (-7.29, -1.28)	0.007	0.114
≥0.05	-2.11 (-4.85, 0.64)	0.134	0.1581	-1.92 (-4.62, 0.78)	0.168	0.2965
Vitality						
WHR	-2.83 (-5.44, -0.21)	0.036	0.09	-1.57 (-3.32, 0.19)	0.085	0.195
WHR levels						
<0.04	Ref			Ref		
0.04-0.05	-4.60 (-9.93, 0.73)	0.093	0.1268	-5.45 (-10.35, -0.55)	0.033	0.1414
≥0.05	-5.42 (-11.22, 0.38)	0.069	0.1035	-5.38 (-9.79, -0.97)	0.019	0.114
Social functioning						
WHR	-2.58 (-5.22, 0.05)	0.056	0.0988	-0.78 (-3.45, 1.90)	0.571	0.6344
WHR levels						
<0.04	Ref			Ref		
0.04-0.05	-5.50 (-10.83, -0.17)	0.045	0.09	-7.57 (-15.05, -0.08)	0.051	0.17
≥0.05	-5.50 (-11.30, 0.30)	0.065	0.1035	-4.06 (-10.79, 2.67)	0.241	0.3443
Role-emotional						
WHR	-2.78 (-5.42, -0.14)	0.040	0.09	-3.41 (-5.88, -0.93)	0.009	0.114
WHR levels						
<0.04	Ref			Ref		
0.04-0.05	-3.54 (-9.25, 2.17)	0.226	0.2421	-7.28 (-14.48, -0.09)	0.051	0.17
≥0.05	-4.74 (-10.96, 1.47)	0.137	0.1581	-5.67 (-12.14, 0.81)	0.091	0.195
Mental health						
WHR	-1.83 (-4.09, 0.42)	0.114	0.1425	-0.73 (-2.55, 1.09)	0.435	0.5019
WHR levels						
<0.04	Ref			Ref		
0.04-0.05	-5.47 (-10.00, -0.94)	0.019	0.0633	-4.77 (-9.88, 0.35)	0.072	0.195
≥0.05	-4.66 (-9.60, 0.27)	0.066	0.1035	-2.89 (-7.48, 1.71)	0.223	0.3443

WHR, white blood cell to hemoglobin ratio; CI, confidence interval; PCS, Physical Health Score; Ref; reference; MCS, Mental Health Score.

**Table 4:** Association between WHR and life quality in BMI subgroups.

Variables	BMI: <24			BMI: ≥24		
	β (95% CI)	*P*	*q*	β (95% CI)	*P*	*q*
PCS						
WHR	-1.46 (-2.33, -0.58)	0.001	0.0075	-2.09 (-3.79, -0.39)	0.021	0.1575
WHR levels						
<0.04						
0.04-0.05	-2.53 (-4.45, -0.62)	0.010	0.0429	3.69 (-1.70, 9.09)	0.189	0.3142
≥0.05	-2.74 (-4.73, -0.75)	0.008	0.0429	-2.16 (-6.28, 1.96)	0.312	0.4255
Physical functioning						
WHR	-2.76 (-4.12, -1.41)	<0.001	0.0075	-4.22 (-7.32, -1.12)	0.011	0.1575
WHR levels						
<0.04	Ref			Ref		
0.04-0.05	-3.78 (-6.75, -0.81)	0.013	0.0487	5.44 (-4.30, 15.19)	0.281	0.4129
≥0.05	-5.25 (-8.34, -2.16)	0.001	0.0075	-6.37 (-13.81, 1.07)	0.102	0.3027
Role-physical						
WHR	-2.10 (-4.08, -0.13)	0.038	0.0814	-5.59 (-9.32, -1.87)	0.006	0.1575
WHR levels						
<0.04	Ref			Ref		
0.04-0.05	-5.64 (-9.91, -1.37)	0.010	0.0429	3.58 (-8.96, 16.12)	0.579	0.6589
≥0.05	-4.42 (-8.86, 0.02)	0.052	0.0821	-6.61 (-16.18, 2.96)	0.185	0.3142
Bodily pain						
WHR	-1.96 (-3.89, -0.02)	0.049	0.0821	-0.17 (-3.71, 3.38)	0.927	0.927
WHR levels						
<0.04	Ref			Ref		
0.04-0.05	-4.92 (-9.12, -0.73)	0.023	0.069	3.73 (-7.41, 14.88)	0.516	0.6192
≥0.05	-3.48 (-7.85, 0.89)	0.120	0.144	3.54 (-4.97, 12.04)	0.421	0.5262
General health						
WHR	-2.09 (-3.98, -0.19)	0.032	0.0814	-3.01 (-5.78, -0.24)	0.040	0.24
WHR levels						
<0.04	Ref			Ref		
0.04-0.05	-3.50 (-7.63, 0.63)	0.099	0.1238	0.68 (-8.31, 9.67)	0.883	0.9134
≥0.05	-3.01 (-7.31, 1.29)	0.171	0.178	-4.80 (-11.66, 2.06)	0.179	0.3142
MCS						
WHR	-0.65 (-1.58, 0.29)	0.178	0.178	-1.44 (-3.29, 0.42)	0.139	0.3107
WHR levels						
<0.04	Ref			Ref		
0.04-0.05	-2.07 (-4.09, -0.05)	0.046	0.0821	-5.10 (-10.89, 0.69)	0.093	0.3027
≥0.05	-1.57 (-3.67, 0.54)	0.146	0.1685	-3.69 (-8.11, 0.73)	0.111	0.3027
Vitality						
WHR	-2.05 (-3.97, -0.13)	0.038	0.0814	-3.76 (-6.78, -0.75)	0.020	0.1575
WHR levels						
<0.04	Ref			Ref		
0.04-0.05	-4.21 (-8.38, -0.03)	0.050	0.0821	-5.44 (-15.34, 4.45)	0.289	0.4129
≥0.05	-4.66 (-9.01, -0.32)	0.037	0.0814	-6.98 (-14.54, 0.57)	0.079	0.2963
Social functioning						
WHR	-1.40 (-3.39, 0.59)	0.169	0.178	-4.57 (-9.25, 0.12)	0.064	0.2743
WHR levels						
<0.04	Ref			Ref		
0.04-0.05	-3.93 (-8.24, 0.37)	0.075	0.1023	-11.59 (-26.32, 3.14)	0.132	0.3107
≥0.05	-3.09 (-7.57, 1.39)	0.178	0.178	-11.57 (-22.81, -0.33)	0.052	0.26
Role-emotional						
WHR	-4.35 (-6.52, -2.19)	<0.001	0.0075	-2.07 (-5.18, 1.03)	0.199	0.3142
WHR levels						
<0.04	Ref			Ref		
0.04-0.05	-4.66 (-9.47, 0.15)	0.059	0.0885	1.04 (-8.98, 11.07)	0.839	0.8989
≥0.05	-5.10 (-10.10, -0.09)	0.047	0.0821	-2.11 (-9.75, 5.54)	0.593	0.6589
Mental health						
WHR	-1.47 (-3.15, 0.20)	0.086	0.1122	-2.33 (-5.39, 0.74)	0.145	0.3107
WHR levels						
<0.04	Ref			Ref		
0.04-0.05	-4.50 (-8.11, -0.88)	0.016	0.0533	-4.24 (-14.04, 5.55)	0.402	0.5243
≥0.05	-3.46 (-7.22, 0.29)	0.072	0.1023	-5.08 (-12.55, 2.40)	0.192	0.3142

WHR, white blood cell to hemoglobin ratio; BMI, body mass index; CI, confidence interval; PCS, Physical Health Score; Ref; reference; MCS, Mental Health Score.

**Table 5:** Association between WHR and life quality in clinical stage subgroups.

Variables	Stage I/II/III			Stage IV		
	β (95% CI)	*P*	*q*	β (95% CI)	*P*	*q*
PCS						
WHR	-1.18 (-2.23, -0.14)	0.027	0.29	-2.02 (-3.26, -0.78)	0.002	0.02
WHR levels						
<0.04						
0.04-0.05	-1.27 (-3.87, 1.32)	0.338	0.4225	-2.05 (-5.02, 0.92)	0.179	0.2148
≥0.05	-2.31 (-4.81, 0.20)	0.073	0.2937	-2.88 (-5.76, 0.00)	0.053	0.106
Physical functioning						
WHR	-2.28 (-4.01, -0.56)	0.011	0.29	-3.91 (-5.79, -2.02)	<0.001	0.02
WHR levels						
<0.04	Ref			Ref		
0.04-0.05	-2.92 (-7.22, 1.38)	0.185	0.2937	-1.66 (-6.26, 2.94)	0.481	0.481
≥0.05	-4.69 (-8.84, -0.53)	0.029	0.29	-5.89 (-10.36, -1.42)	0.011	0.066
Role-physical						
WHR	-2.13 (-4.57, 0.31)	0.090	0.2937	-2.68 (-5.24, -0.12)	0.043	0.1038
WHR levels						
<0.04	Ref			Ref		
0.04-0.05	-4.28 (-10.29, 1.74)	0.166	0.2937	-3.80 (-9.85, 2.26)	0.222	0.2467
≥0.05	-5.94 (-11.74, -0.13)	0.047	0.2937	-2.34 (-8.21, 3.53)	0.437	0.4521
Bodily pain						
WHR	-0.94 (-3.16, 1.29)	0.412	0.4811	-2.99 (-5.59, -0.38)	0.027	0.084
WHR levels						
<0.04	Ref			Ref		
0.04-0.05	-2.14 (-7.66, 3.38)	0.449	0.4811	-7.16 (-13.21, -1.11)	0.022	0.084
≥0.05	-0.99 (-6.32, 4.34)	0.716	0.716	-4.95 (-10.81, 0.92)	0.102	0.1457
General health						
WHR	-1.67 (-3.66, 0.31)	0.101	0.2937	-2.44 (-5.18, 0.31)	0.085	0.132
WHR levels						
<0.04	Ref			Ref		
0.04-0.05	-1.15 (-5.94, 3.63)	0.638	0.66	-4.34 (-10.77, 2.10)	0.190	0.2192
≥0.05	-3.15 (-7.77, 1.48)	0.185	0.2937	-3.35 (-9.60, 2.89)	0.295	0.3161
MCS						
WHR	-0.45 (-1.53, 0.64)	0.422	0.4811	-1.37 (-2.56, -0.18)	0.027	0.084
WHR levels						
<0.04	Ref			Ref		
0.04-0.05	-2.06 (-4.72, 0.61)	0.133	0.2937	-2.92 (-5.70, -0.15)	0.042	0.1038
≥0.05	-1.75 (-4.33, 0.83)	0.186	0.2937	-2.37 (-5.06, 0.32)	0.088	0.132
Vitality						
WHR	-1.53 (-3.65, 0.58)	0.157	0.2937	-3.57 (-6.13, -1.01)	0.008	0.06
WHR levels						
<0.04	Ref			Ref		
0.04-0.05	-2.03 (-7.25, 3.19)	0.447	0.4811	-6.03 (-12.06, 0.00)	0.053	0.106
≥0.05	-4.16 (-9.20, 0.88)	0.108	0.2937	-6.07 (-11.92, -0.22)	0.045	0.1038
Social functioning						
WHR	-1.48 (-3.87, 0.91)	0.229	0.3271	-3.13 (-5.81, -0.45)	0.024	0.084
WHR levels						
<0.04	Ref			Ref		
0.04-0.05	-4.73 (-10.62, 1.15)	0.118	0.2937	-5.94 (-12.21, 0.33)	0.066	0.1117
≥0.05	-4.31 (-9.99, 1.38)	0.140	0.2937	-5.74 (-11.82, 0.34)	0.067	0.1117
Role-emotional						
WHR	-2.15 (-4.68, 0.39)	0.099	0.2937	-4.40 (-7.07, -1.73)	0.002	0.02
WHR levels						
<0.04	Ref			Ref		
0.04-0.05	-4.05 (-10.42, 2.33)	0.216	0.324	-5.17 (-11.58, 1.24)	0.117	0.1487
≥0.05	-3.65 (-9.82, 2.51)	0.247	0.3368	-6.11 (-12.32, 0.11)	0.057	0.1069
Mental health						
WHR	-0.97 (-2.91, 0.96)	0.326	0.4225	-2.48 (-4.65, -0.30)	0.028	0.084
WHR levels						
<0.04	Ref			Ref		
0.04-0.05	-3.96 (-8.73, 0.80)	0.105	0.2937	-4.10 (-9.21, 1.00)	0.119	0.1487
≥0.05	-3.33 (-7.93, 1.28)	0.159	0.2937	-4.11 (-9.06, 0.85)	0.108	0.1473

WHR, white blood cell to hemoglobin ratio; CI, confidence interval; PCS, Physical Health Score; Ref; reference; MCS, Mental Health Score.

**Table 6:** Association between WHR and life quality in pathological classification subgroups.

Variables	Ovary/peritoneal			Ovary/peritoneal combined with deep infiltration		
	β (95% CI)	*P*	*q*	β (95% CI)	*P*	*q*
PCS						
WHR	-1.33 (-2.10, -0.55)	0.001	0.015	-1.29 (-3.20, 0.61)	0.189	0.3568
WHR levels						
<0.04	Ref			Ref		
0.04-0.05	-0.57 (-2.48, 1.35)	0.563	0.6032	-3.75 (-8.20, 0.69)	0.103	0.315
≥0.05	-2.24 (-4.06, -0.42)	0.017	0.1275	-2.03 (-6.61, 2.55)	0.388	0.4752
Physical functioning						
WHR	-2.51 (-3.77, -1.26)	<0.001	0.015	-2.83 (-5.83, 0.17)	0.069	0.3
WHR levels						
<0.04	Ref			Ref		
0.04-0.05	-1.58 (-4.68, 1.51)	0.317	0.3962	-4.43 (-11.54, 2.68)	0.226	0.3568
≥0.05	-4.78 (-7.71, -1.85)	0.002	0.02	-4.87 (-12.19, 2.44)	0.196	0.3568
Role-physical						
WHR	-1.86 (-3.92, 0.21)	0.080	0.244	-2.17 (-5.62, 1.28)	0.223	0.3568
WHR levels						
<0.04	Ref			Ref		
0.04-0.05	-3.18 (-8.20, 1.84)	0.216	0.324	-6.30 (-14.36, 1.76)	0.130	0.325
≥0.05	-4.44 (-9.19, 0.31)	0.069	0.244	-1.79 (-10.08, 6.51)	0.674	0.6972
Bodily pain						
WHR	-1.50 (-3.33, 0.33)	0.111	0.244	-1.69 (-5.45, 2.07)	0.381	0.4752
WHR levels						
<0.04	Ref			Ref		
0.04-0.05	-0.59 (-5.07, 3.89)	0.796	0.806	-11.15 (-19.58, -2.72)	0.012	0.18
≥0.05	-1.30 (-5.55, 2.95)	0.550	0.6032	-3.19 (-11.88, 5.49)	0.474	0.5469
General health						
WHR	-1.49 (-3.00, 0.02)	0.054	0.244	-1.11 (-5.12, 2.90)	0.589	0.6311
WHR levels						
<0.04	Ref			Ref		
0.04-0.05	-0.48 (-4.29, 3.33)	0.806	0.806	-7.72 (-16.94, 1.49)	0.105	0.315
≥0.05	-2.87 (-6.47, 0.74)	0.122	0.244	-1.00 (-10.49, 8.50)	0.838	0.838
MCS						
WHR	-0.36 (-1.23, 0.52)	0.427	0.5124	-1.20 (-3.10, 0.70)	0.219	0.3568
WHR levels						
<0.04	Ref			Ref		
0.04-0.05	-1.48 (-3.60, 0.63)	0.172	0.2867	-5.33 (-9.64, -1.01)	0.018	0.18
≥0.05	-1.48 (-3.49, 0.53)	0.150	0.2812	-1.94 (-6.38, 2.51)	0.396	0.4752
Vitality						
WHR	-1.43 (-3.15, 0.29)	0.105	0.244	-3.13 (-6.86, 0.59)	0.104	0.315
WHR levels						
<0.04	Ref			Ref		
0.04-0.05	-1.57 (-5.75, 2.61)	0.462	0.5331	-9.92 (-18.52, -1.32)	0.027	0.18
≥0.05	-3.61 (-7.58, 0.35)	0.076	0.244	-5.92 (-14.77, 2.94)	0.195	0.3568
Social functioning						
WHR	-1.03 (-2.91, 0.86)	0.287	0.3743	-3.02 (-7.22, 1.18)	0.163	0.3568
WHR levels						
<0.04	Ref			Ref		
0.04-0.05	-3.20 (-7.75, 1.35)	0.170	0.2867	-10.93 (-20.58, -1.29)	0.030	0.18
≥0.05	-3.77 (-8.08, 0.54)	0.088	0.244	-4.80 (-14.74, 5.13)	0.347	0.4752
Role-emotional						
WHR	-2.50 (-4.61, -0.38)	0.022	0.132	-3.08 (-6.96, 0.80)	0.124	0.325
WHR levels						
<0.04	Ref			Ref		
0.04-0.05	-3.29 (-8.68, 2.11)	0.234	0.33	-8.48 (-17.53, 0.56)	0.070	0.3
≥0.05	-4.08 (-9.20, 1.03)	0.119	0.244	-4.38 (-13.70, 4.93)	0.360	0.4752
Mental health						
WHR	-0.99 (-2.52, 0.54)	0.208	0.324	-1.64 (-5.15, 1.87)	0.362	0.4752
WHR levels						
<0.04	Ref			Ref		
0.04-0.05	-2.23 (-5.94, 1.49)	0.242	0.33	-9.05 (-17.03, -1.07)	0.030	0.18
≥0.05	-2.89 (-6.42, 0.63)	0.109	0.244	-2.76 (-10.99, 5.46)	0.513	0.57

WHR, white blood cell to hemoglobin ratio; CI, confidence interval; PCS, Physical Health Score; Ref; reference; MCS, Mental Health Score.

**Table 7:** Association between WHR and life quality in pelvic pain subgroups.

Variables	Pelvic pain: No			Pelvic pain: Yes		
	β (95% CI)	*P*	*q*	β (95% CI)	*P*	*q*
PCS						
WHR	-1.66 (-2.35, -0.98)	**<0.001**	0.015	-0.81 (-2.85, 1.24)	0.444	0.6343
WHR levels						
<0.04	Ref			Ref		
0.04-0.05	-0.27 (-1.85, 1.31)	0.739	0.739	-7.54 (-12.60, -2.47)	0.005	0.0375
≥0.05	-2.24 (-3.82, -0.65)	0.006	0.0257	-3.14 (-8.20, 1.92)	0.229	0.5057
Physical functioning						
WHR	-3.20 (-4.42, -1.97)	**<0.001**	0.015	-1.90 (-5.01, 1.21)	0.236	0.5057
WHR levels						
<0.04	Ref			Ref		
0.04-0.05	0.58 (-2.24, 3.40)	0.686	0.7097	-12.24 (-19.93, -4.56)	0.003	0.03
≥0.05	-4.35 (-7.18, -1.52)	0.003	0.015	-6.95 (-14.62, 0.72)	0.080	0.24
Role-physical						
WHR	-2.95 (-4.75, -1.15)	**0.002**	0.015	-0.44 (-4.43, 3.55)	0.830	0.8669
WHR levels						
<0.04	Ref			Ref		
0.04-0.05	-2.35 (-6.41, 1.71)	0.259	0.2988	-11.03 (-21.08, -0.98)	0.035	0.1167
≥0.05	-4.59 (-8.66, -0.52)	0.029	0.0725	-1.77 (-11.80, 8.27)	0.731	0.8669
Bodily pain						
WHR	-2.28 (-3.67, -0.89)	**0.002**	0.015	0.66 (-3.67, 4.98)	0.766	0.8669
WHR levels						
<0.04	Ref			Ref		
0.04-0.05	-3.03 (-6.18, 0.11)	0.061	0.1076	-13.42 (-24.15, -2.69)	0.017	0.0675
≥0.05	-2.99 (-6.14, 0.16)	0.065	0.1083	-1.37 (-12.09, 9.35)	0.803	0.8669
General health						
WHR	-0.98 (-2.19, 0.23)	0.116	0.1657	-2.06 (-6.33, 2.21)	0.348	0.58
WHR levels						
<0.04	Ref			Ref		
0.04-0.05	-0.68 (-3.58, 2.22)	0.647	0.6932	-13.87 (-24.61, -3.13)	0.014	0.0675
≥0.05	-2.11 (-5.02, 0.80)	0.158	0.1975	-5.78 (-16.51, 4.94)	0.294	0.5188
MCS						
WHR	-0.57 (-1.30, 0.16)	0.128	0.1735	-0.51 (-2.55, 1.54)	0.629	0.7863
WHR levels						
<0.04	Ref			Ref		
0.04-0.05	-1.47 (-3.07, 0.14)	0.075	0.1184	-7.22 (-12.27, -2.17)	0.007	0.042
≥0.05	-1.69 (-3.30, -0.08)	0.042	0.09	-2.21 (-7.25, 2.84)	0.394	0.591
Vitality						
WHR	-1.93 (-3.35, -0.50)	**0.009**	0.03	-1.90 (-6.07, 2.28)	0.377	0.591
WHR levels						
<0.04	Ref			Ref		
0.04-0.05	-2.43 (-5.58, 0.72)	0.133	0.1735	-13.09 (-23.62, -2.55)	0.018	0.0675
≥0.05	-4.40 (-7.55, -1.24)	0.007	0.0262	-5.93 (-16.45, 4.58)	0.273	0.5188
Social functioning						
WHR	-1.87 (-3.45, -0.29)	**0.022**	0.06	-1.48 (-5.99, 3.03)	0.523	0.7132
WHR levels						
<0.04	Ref			Ref		
0.04-0.05	-2.06 (-5.58, 1.47)	0.255	0.2988	-18.61 (-29.56, -7.66)	0.001	0.03
≥0.05	-3.72 (-7.25, -0.19)	0.041	0.09	-7.70 (-18.64, 3.23)	0.172	0.43
Role-emotional						
WHR	-2.13 (-3.51, -0.75)	**0.003**	0.015	-0.50 (-5.31, 4.30)	0.838	0.8669
WHR levels						
<0.04	Ref			Ref		
0.04-0.05	-3.06 (-6.51, 0.38)	0.083	0.1245	-9.81 (-22.09, 2.48)	0.122	0.3327
≥0.05	-4.42 (-7.87, -0.97)	0.013	0.039	-0.82 (-13.08, 11.45)	0.896	0.896
Mental health						
WHR	-1.38 (-2.76, 0.00)	0.051	0.0956	-1.02 (-4.63, 2.59)	0.581	0.7578
WHR levels						
<0.04	Ref			Ref		
0.04-0.05	-1.64 (-4.70, 1.41)	0.293	0.3256	-14.66 (-23.41, -5.91)	0.002	0.03
≥0.05	-3.08 (-6.15, -0.02)	0.050	0.0956	-4.82 (-13.56, 3.91)	0.283	0.5188

WHR, white blood cell to hemoglobin ratio; CI, confidence interval; PCS, Physical Health Score; Ref; reference; MCS, Mental Health Score.

Bold values indicate statistical significance at the 2-sided P < 0.05.

**Table 8:** Association between WHR and life quality in lysis of adhesion subgroups.

Variables	Only left/right adhesion			Left/right adhesion combined with rectum-vagina		
	β (95% CI)	*P*	*q*	β (95% CI)	*P*	*q*
PCS						
WHR	-1.35 (-2.53, -0.18)	0.026	0.168	-1.84 (-2.97, -0.71)	0.002	0.02
WHR levels						
<0.04	Ref			Ref		
0.04-0.05	-2.76 (-5.65, 0.12)	0.064	0.2588	-1.99 (-4.49, 0.50)	0.120	0.1385
≥0.05	-3.13 (-5.88, -0.38)	0.028	0.168	-2.84 (-5.39, -0.29)	0.031	0.06
Physical functioning						
WHR	-2.69 (-4.61, -0.76)	0.008	0.12	-3.67 (-5.47, -1.86)	<0.001	0.02
WHR levels						
<0.04	Ref			Ref		
0.04-0.05	-5.40 (-10.13, -0.68)	0.028	0.168	-2.09 (-6.10, 1.92)	0.309	0.309
≥0.05	-6.35 (-10.84, -1.85)	0.007	0.12	-5.78 (-9.88, -1.68)	0.007	0.035
Role-physical						
WHR	-2.41 (-5.47, 0.65)	0.126	0.3035	-2.68 (-4.97, -0.38)	0.024	0.0554
WHR levels						
<0.04	Ref			Ref		
0.04-0.05	-6.10 (-13.58, 1.39)	0.114	0.3035	-4.09 (-9.11, 0.92)	0.112	0.1344
≥0.05	-6.70 (-13.82, 0.42)	0.069	0.2588	-3.47 (-8.60, 1.66)	0.187	0.2004
Bodily pain						
WHR	-0.03 (-2.64, 2.58)	0.981	0.981	-2.97 (-5.27, -0.66)	0.013	0.0382
WHR levels						
<0.04	Ref			Ref		
0.04-0.05	-4.48 (-10.85, 1.89)	0.172	0.3035	-6.55 (-11.53, -1.57)	0.011	0.0367
≥0.05	-0.18 (-6.24, 5.88)	0.953	0.981	-5.45 (-10.53, -0.36)	0.038	0.0671
General health						
WHR	-2.30 (-4.60, -0.01)	0.052	0.2588	-1.64 (-3.98, 0.70)	0.171	0.19
WHR levels						
<0.04	Ref			Ref		
0.04-0.05	-1.76 (-7.75, 4.22)	0.565	0.6467	-5.01 (-10.05, 0.02)	0.053	0.0723
≥0.05	-4.75 (-10.45, 0.95)	0.106	0.3035	-2.76 (-7.91, 2.39)	0.296	0.3062
MCS						
WHR	-0.22 (-1.53, 1.10)	0.749	0.8025	-1.17 (-2.34, 0.00)	0.052	0.0723
WHR levels						
<0.04	Ref			Ref		
0.04-0.05	-1.54 (-4.78, 1.69)	0.352	0.48	-3.80 (-6.29, -1.32)	0.003	0.0225
≥0.05	-1.56 (-4.64, 1.51)	0.322	0.46	-2.48 (-5.03, 0.06)	0.058	0.0757
Vitality						
WHR	-1.57 (-4.41, 1.28)	0.283	0.4245	-2.89 (-5.08, -0.71)	0.010	0.0367
WHR levels						
<0.04	Ref			Ref		
0.04-0.05	-1.98 (-8.97, 5.02)	0.582	0.6467	-6.40 (-11.12, -1.69)	0.009	0.0367
≥0.05	-4.72 (-11.38, 1.94)	0.168	0.3035	-5.38 (-10.20, -0.56)	0.030	0.06
Social functioning						
WHR	-1.08 (-4.10, 1.95)	0.488	0.61	-2.76 (-5.26, -0.26)	0.032	0.06
WHR levels						
<0.04	Ref			Ref		
0.04-0.05	-4.59 (-11.99, 2.80)	0.227	0.3584	-7.69 (-13.04, -2.35)	0.005	0.03
≥0.05	-5.35 (-12.38, 1.69)	0.140	0.3035	-5.64 (-11.11, -0.17)	0.045	0.0723
Role-emotional						
WHR	-2.07 (-4.97, 0.84)	0.167	0.3035	-4.14 (-6.67, -1.61)	0.002	0.02
WHR levels						
<0.04	Ref			Ref		
0.04-0.05	-5.34 (-12.85, 2.17)	0.167	0.3035	-5.71 (-11.27, -0.15)	0.046	0.0723
≥0.05	-2.36 (-9.50, 4.79)	0.520	0.624	-6.76 (-12.44, -1.07)	0.021	0.0525
Mental health						
WHR	-0.79 (-2.90, 1.32)	0.467	0.6091	-2.21 (-4.42, 0.00)	0.052	0.0723
WHR levels						
<0.04	Ref			Ref		
0.04-0.05	-3.63 (-8.79, 1.53)	0.171	0.3035	-6.04 (-10.78, -1.30)	0.014	0.0382
≥0.05	-3.24 (-8.14, 1.67)	0.200	0.3333	-4.65 (-9.50, 0.20)	0.062	0.0775

WHR, white blood cell to hemoglobin ratio; CI, confidence interval; PCS, Physical Health Score; Ref; reference; MCS, Mental Health Score.

Note on multiplicity (applies to subgroup tables): P-values have been adjusted within each table using the Benjamini–Hochberg procedure to control the false discovery rate (FDR); adjusted values are shown as “q=” appended to P. Significance for subgroups was defined as q<0.05 (q<0.10 considered suggestive).

According to **Table 2**, an elevated WHR was associated with decreased scores of PCS (β=-1.42, 95%CI: -2.20, -0.65, *P* < 0.001), physical functioning (β=-2.82, 95%CI: -4.06, -1.58, *P* < 0.001), role-physical (β=-2.21, 95%CI: -3.94, -0.49, *P* = 0.012), vitality (β=-2.00, 95%CI: -3.59, -0.41, *P* = 0.014) and role-emotional (β=-2.41, 95%CI: -4.23, -0.59, *P* = 0.010) in SF-36 scale, after adjusting for all selected covariates. Compared to WHR level of <0.04, EM patients with WHR level of [0.04-0.05) or ≥0.05 had decreased scores of PCS, physical functioning, role-physical, vitality, social functioning, role-emotional and mental health (raw *P* < 0.05). Also, associations of WHR level of [0.04-0.05) with decreased scores of bodily pain (β=-5.26, 95%CI: -9.14, -1.38, *P* = 0.008), general health (β=-4.00, 95%CI: -7.73, -0.28, *P* = 0.036) and MCS (β=-2.78, 95%CI: -4.66, -0.90, *P* = 0.004) were significant, compared to WHR level of <0.04.

### Association between WHR and the quality of life in different subgroups

Prespecified subgroup analyses (age, BMI, rASRM stage, phenotype, pelvic pain, adhesion type) showed directionally consistent associations. After Benjamini–Hochberg FDR control within each subgroup family, the inverse WHR–PCS association remained significant in age<40, BMI<24 kg/m², rASRM stage IV, ovary/peritoneal phenotype, pelvic pain: no, and left/right adhesion combined with rectum–vaginal adhesion (q<0.05), whereas other contrasts did not survive multiplicity adjustment and should be interpreted as exploratory (**Tables 3**-**8**). Domain-level patterns were similar but attenuated after FDR, with q<0.05 observed mainly in PF, BP, VT, RP and RE within selected strata.

## Discussion

In this single-center cohort of women undergoing laparoscopic surgery for endometriosis (EM), higher pre-operative white-blood-cell-to-hemoglobin ratio (WHR)—a composite reflecting systemic inflammation (WBC) and anemia/oxygen-carrying capacity (Hb)—was associated with lower postoperative health-related quality of life (HRQoL) at 3 months, particularly the Physical Component Summary (PCS) and the domains of physical functioning, role-physical, vitality and role-emotional, after covariate adjustment. Tertile groupings were used only for description, whereas primary inference treated WHR as a continuous exposure, given the absence of disease-specific clinical cut-offs for EM and non-transportability of oncology thresholds with differing unit conventions.

Mechanistic considerations. The observed associations are biologically plausible and now supported by converging clinical and experimental evidence. First, EM features chronic low-grade inflammation with altered peritoneal immune milieu and cytokine signaling that track pain and symptom burden (5) (14, 15). Inflammation is also a robust prognostic signal for adverse surgical outcomes broadly (6). Second, anemia is common perioperatively and in EM (e.g., menorrhagia/iron loss), and is itself linked to worse recovery, fatigue and functional limitation (7, 8). Mechanistically, inflammation drives hepcidin-mediated iron sequestration (anemia of inflammation), reducing Hb and tissue oxygen delivery (16). Third, lower Hb and impaired oxygen transport correlate with poorer SF-36 functioning and pain in other chronic populations, supporting a pathway from anemia to reduced physical and social functioning (17). Fourth, inflammation and oxidative stress engage neuroimmune circuits (microglial activation, mitochondrial dysfunction, neurotransmitter alterations) that are implicated in fatigue, low vitality and depressive symptoms (18) (19) (20) (21). Together, these data outline a dual-axis model—inflammation (↑WBC) and anemia (↓Hb)—by which higher WHR could plausibly contribute to lower postoperative HRQoL in EM via impaired oxygen delivery, neuroinflammation, and sensitization of pain-affect networks. While our observational design precludes causal inference, these independent strands of clinical and mechanistic literature provide a coherent framework consistent with our findings.

Clinical applicability and subgroups. We envision WHR as a complementary risk indicator—not a stand-alone rule—to help flag patients at higher risk of poorer physical recovery at 3 months. In our prespecified subgroup analyses (age, BMI, rASRM stage, phenotype, pelvic pain, adhesion type), patterns were broadly consistent; after false-discovery-rate control, the inverse WHR-PCS association persisted in selected strata, supporting internal consistency of the signal, whereas other contrasts did not survive multiplicity adjustment and should be viewed as exploratory (see **Tables 3**-**8** for raw P and q). In practice, WHR may be most informative when integrated with advanced stage, ovarian/peritoneal phenotype, or anemia risk (e.g., heavy bleeding/iron deficiency (8) (16)) and symptom profiles where pain is less dominant (consistent with peritoneal immune correlates of pain rather than lesion volume (14) (15)). Clinically actionable steps could include: (i) targeted pre-/post-operative anemia assessment and iron studies in high-WHR patients; (ii) optimization of anti-inflammatory and hormonal management per guidelines; and (iii) closer HRQoL follow-up and rehabilitation emphasis on fatigue and physical function domains. These proposals are hypothesis-generating and require prospective testing.

MCID and interpretation of effect sizes. To aid clinical interpretation, we considered distribution-based anchors (~0.5 SD) commonly used in HRQoL research to approximate the minimal clinically important difference (MCID) for SF-36 subscales (16) (22) (23) (24). Against this benchmark, the observed decrements in physical functioning and vitality with higher WHR may approach clinical importance in aggregate, even if mean differences are modest. Because MCIDs can vary by population, baseline severity and domain, we refrain from asserting a single numeric cut-off and instead present β estimates with 95% CIs alongside this anchor to facilitate transparent, context-specific interpretation.

Multiplicity, precision and residual confounding. Multiple subgroup contrasts increase the risk of false positives; we therefore controlled the false discovery rate (Benjamini–Hochberg) and emphasize effect sizes and precision over dichotomous significance. After adjustment, false negatives remain possible given limited subgroup sizes. Although we adjusted for key clinical factors, residual confounding (e.g., postoperative hormonal suppression, iron-deficiency status, baseline pain) cannot be excluded, and findings should be interpreted as associational.

Strengths, limitations and next steps. Strengths include a novel focus on EM HRQoL, a comprehensive validated instrument (SF-36) (16) (22) (23) (24), prespecified modelling, and minimal missingness with consistent complete-case and imputed results (**Supplementary Tables S1**-**S2**). Limitations include the retrospective single-center design and potential referral bias (higher proportion of advanced disease), which may constrain generalizability. Prospective, multicenter studies with repeated HRQoL measures, granular iron indices and inflammatory markers, and derivation/validation of EM-specific WHR thresholds are warranted to confirm clinical utility and define for whom WHR-guided pathways add the most value.

## Data Availability

The original contributions presented in the study are included in the article/**Supplementary Material**. Further inquiries can be directed to the corresponding authors.
