# Supplementary material for: Association of white blood cell count to hemoglobin ratio with the life quality after laparoscopic surgery in patients with endometriosis
Source: Front Endocrinol (Lausanne). 2025 Oct 13;16:1655476. doi: 10.3389/fendo.2025.1655476 (PMC12554603; doi:10.3389/fendo.2025.1655476)
Supplement: Supplementary file 1 [file DataSheet1.docx]

**Supplementary Methods**

Causal assumptions and identification strategy

We conceptualised the association between the pre-operative white-blood-cell-to-haemoglobin ratio (WHR) and the 3-month SF-36 Physical Component Summary (PCS) within a directed acyclic graph (DAG; Supplementary Figure). WHR is the exposure and 3-month PCS the outcome. The measured confounders included a priori were: age, BMI, disease severity (rASRM stage), phenotype (ovary/peritoneal), baseline pelvic pain, gynaecologic history (adenomyosis/fibroids), parity, and laboratory covariates (albumin, fibrinogen, AST/ALT, creatinine). These variables were selected based on clinical reasoning and literature linking them to systemic inflammation/anaemia and to quality-of-life outcomes.

Unmeasured common causes that may affect both WHR and PCS include iron deficiency/menorrhagia, baseline mental health, and socioeconomic/lifestyle factors. Post-operative hormonal suppression, post-operative complications, and surgical complexity (e.g., adhesiolysis) are conceptualised as mediators on the pathway from pre-operative biology and disease severity to subsequent quality of life.

Under these assumptions, a sufficient adjustment set to block backdoor paths from WHR to PCS comprises the measured confounders listed above. We acknowledge that including intra-operative/post-operative variables (e.g., adhesiolysis, complications, hormonal suppression) may introduce bias if they lie on the causal pathway (mediators) or act as colliders; accordingly, our primary adjustment set emphasised pre-exposure patient factors and laboratory proxies, and post-operative variables were not included in causal effect estimation. Given residual unmeasured confounding cannot be excluded in an observational study, all effect estimates are interpreted as associations.

Sensitivity considerations. Missingness was minimal and handled via complete-case analysis with multiple imputation as a robustness check; subgroup multiplicity was addressed using Benjamini–Hochberg FDR. Future prospective work with richer measurement of iron indices, menstrual blood loss, baseline pain and mental health, and standardised post-operative hormonal regimens will further strengthen identification and external validity.

**Supplementary Figure**

**
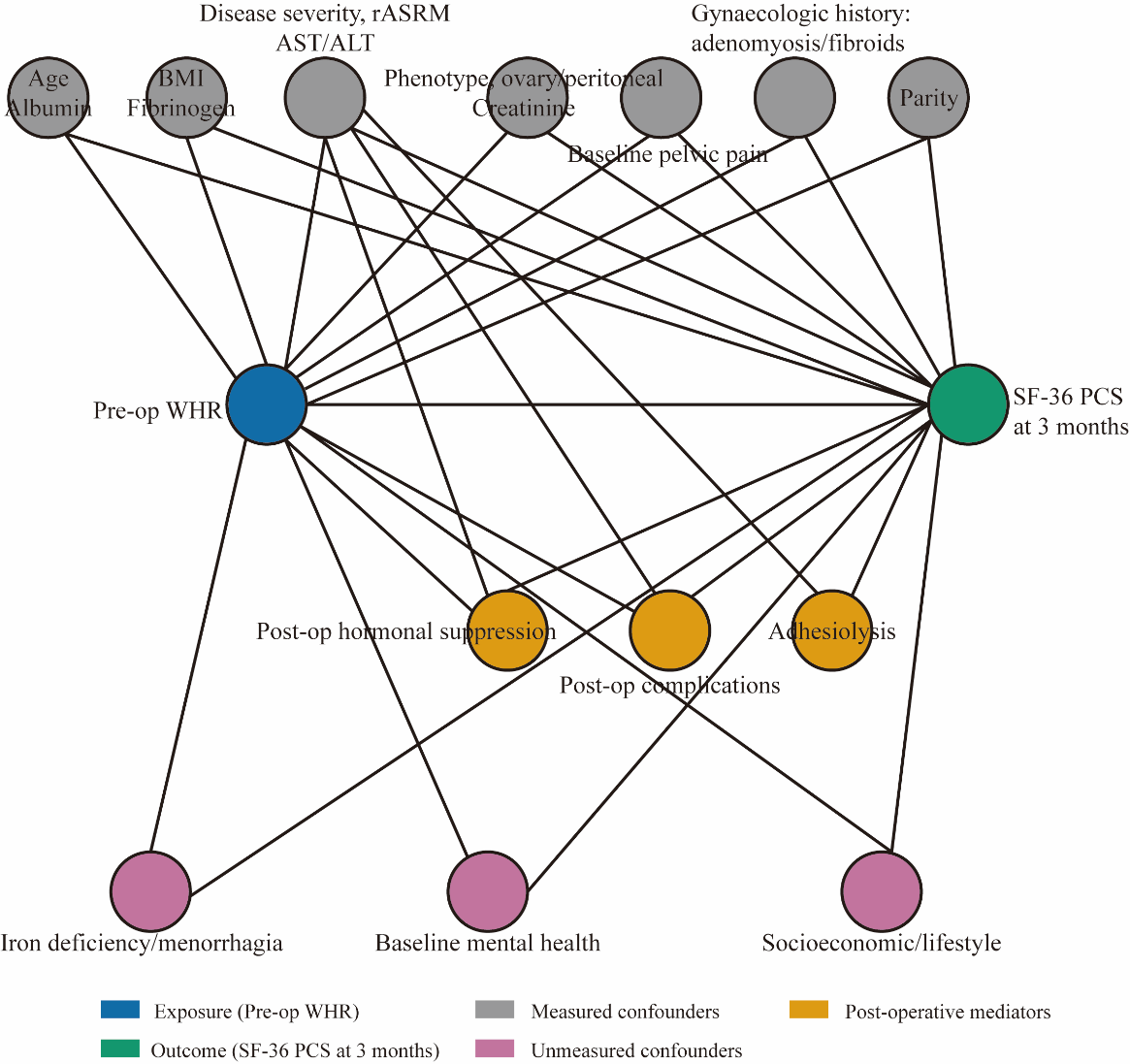
**

Supplementary Figure. Causal directed acyclic graph (DAG). The DAG specifies pre-operative WHR as the exposure and 3-month SF-36 PCS as the outcome. Measured confounders (grey) include age, BMI, rASRM stage, phenotype (ovary/peritoneal), baseline pelvic pain, gynaecologic history (adenomyosis/fibroids), parity, albumin, fibrinogen, AST/ALT, and creatinine. Unmeasured confounders (purple) include iron deficiency/menorrhagia, baseline mental health, and socioeconomic/lifestyle factors. Post-operative hormonal suppression, post-operative complications, and surgical complexity (adhesiolysis) are treated as mediators (orange). The primary adjustment set uses pre-exposure variables; post-operative variables are not adjusted for in causal effect estimation.
