# Supplementary material for: Association of white blood cell count to hemoglobin ratio with the life quality after laparoscopic surgery in patients with endometriosis
Source: Front Endocrinol (Lausanne). 2025 Oct 13;16:1655476. doi: 10.3389/fendo.2025.1655476 (PMC12554603; doi:10.3389/fendo.2025.1655476)
Supplement: Supplementary file 2 [file DataSheet2.docx]

Table S1 Variables’ data with missing values

| Variables | Sample size | Missing frequency (%) |
| --- | --- | --- |
| Cr | 269 | 2 (0.74) |
| ALB | 269 | 2 (0.74) |
| AST/ALT | 269 | 2 (0.74) |

Cr: creatinine, ALB: albumin, AST/ALT: glutamic oxaloacetic transaminase to serum glutamic pyruvic transaminase ratio.

Table S2 Sensitivity analysis on participants’ characteristics before and after multiple interpolation of missing values

| Variables | After multiple interpolation | Before multiple interpolation | Statistics | *P* |
| --- | --- | --- | --- | --- |
| Cr, mg/L Mean ± SD | 55.49 ± 7.91 | 55.45 ± 7.89 | t=0.06 | 0.952 |
| ALB, g/L, Mean ± SD | 44.01 ± 3.45 | 44.02 ± 3.45 | t=-0.04 | 0.970 |
| AST/ALT, M (Q_1_, Q_3_) | 1.60 (1.25, 2.00) | 1.60 (1.25, 2.00) | Z=-0.013 | 0.990 |

Cr: creatinine, ALB: albumin, AST/ALT: glutamic oxaloacetic transaminase to serum glutamic pyruvic transaminase ratio.
